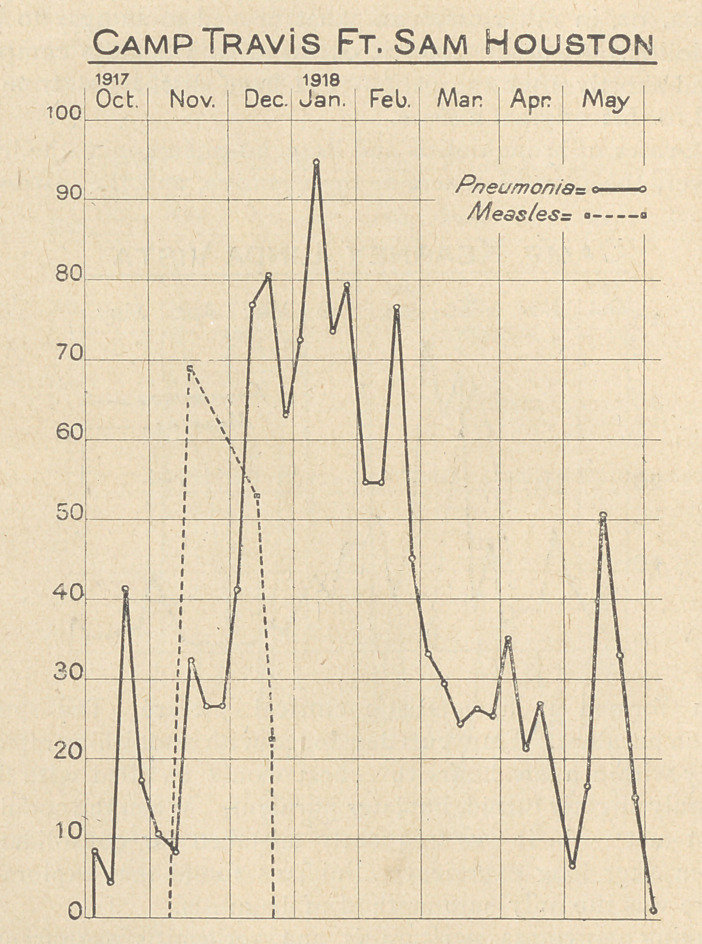# The Relation of the Streptococcus Haemolyticus to Pneumonia in the Troops in the United States

**Published:** 1918-11

**Authors:** Warield Longcope


**The Relation of the Streptococcus Haemolyticus to Pneumonia in the Troops in the United States.** Colonel WARIELD LONGCOPE, M. C.

The speaker said that he would approach the subject from a rather broad point of view, omitting many interesting details, because his information had been gathered from data received from all parts of the country, and only to a small extent from clinical study of individual cases. He said in part:

It was early recognized that pneumonia might be one of the serious diseases which must be dealt with, but I think no one realized how complicated the problem would become nor how difficult to deal with. You will remember that shortly after the first draft was called, and as soon as the men reached the Camps in any large numbers, an epidemic of measles swept through many cantonments and camps, especially those in the South. Pneumonia then appeared first, as a serious factor, and from early in the autumn until late in the spring the respiratory infections were by far the most serious and most common disease in the army.

For several months the exact nature and the true significance of these infections was not known. But shortly after the first of the year, bacteriological work in the Base Hospitals and the investigations of special committees, such as the one headed by Drs. Cole and Mac Callum, and that established at Camp Lee for the investigation of empyema, served to elucidate the etiology of the infections and to point out the way for a study of their control and treatment.

There proved to be two important types of respiratory infection which had to be dealt with — first, lobar pneumonia, caused by the various types of pneumococci; second, an infection of the respiratory tract and serous membranes, caused by the streptococcus haemolyticus.

Though lobar pneumonia was fairly common, the disease on the whole was not very severe, and the mortality was not high. As soon as the facilities and the bacteriologists were available in the cantonment hospitals, the pneumonias were typed in most places, and those due to Type 1 were treated by serum. In the returns, which were received from the Hospitals, ending March 31st, the mortality for this group was low. As I remember it, the figures were 12 to 14 o/o. For the cases due to 2s and 3s, the mortality was much higher.

The pneumococcus pneumonia was, however, entirely overshadowed by the importance of the streptococcus infection. This infection was endemic in certain areas, but occurred as a well defined epidemic, as I will show later, in others. It affected certain groups of individuals.

1st. Patients suffering from measles.

2nd. Patients suffering from pneumococcus lobar pneumonia.

3rd. Patients giving history of a previous mild bronchitis.

4th. Apparently healthy individuals.

As I have previously indicated, pneumonia first appeared as an important disease in the Southern camps in October and November, when it caused a large porportion of the deaths in measles. This was not altogether unexpected, for, as you know, an especially severe form of pneumonia has long been familiar as a complication of measles, and has been greatly feared, especially among troops.

It was also suspected that pneumonia was due to a secondary infection, which spread by contact from one individual to another. The isolation of the individual was impossible. To take its place the ingenious method of separating the measles by means of sheets was devised and immediately put into operation everywhere.

The inical course of these infections, with the extraordinarily high incidence of fatal empyema, as well as the pathology and bacteriology, are familiar to you from the many reports that have already been published. It is only necessary, therefore, to emphasize certain features that were especially striking. In cases of measles, the onset was often insidious and could only be recognized by the rapid development of signs and symptoms of empyema, or, in other cases, of what proved at autopsy to be a bronchiolitis. At other times it occurred as a definite complication, and shortly after the patient had been discharged from the hospital for his original disease.

In the cases of bronchiolitis and broncho-pneumonia, the irregular fever, the marked toxemia, the cyanosis, and the distressing dyspnea formed a characteristic clinical picture. The sputum was often purulent. Empyema developed with astonishing rapidity. Actually within a few hours the chest would be filled with fluid, at first only slightly turbid, but later purulent. When the condition was first met with, it was frequently mistaken for consolidation, and even after long experience clinicians found that exploratory puncture was the only sure method of diagnosis.

Pocketing empyemas, pericarditis, and peritonitis were frequent in some groups. During the early stages of the disease, evidence of intoxication was striking, but in the majority of cases there was no bacteriemia, and blood culture gave no growth. Occasionally, and most commonly after an operation of empyema, metastatic foci of infection appeared in the joints and muscles.

One may well imagine that the presence of this formidable and unusual infection stimulated a tremendous amount of work on the subject. The pathology has been excellently studied, and the problems in bacteriology and immuniology are being investigated by most of the leading bacteriologists in the United States.

It was soon discovered that the infection was introduced into the camps by recruits, and in some regions at least 5 o/o of the men showed streptococcus haemolyticus in their throats upon arrival. The organism evidently spread rapidly from these carriers, particularly where there was crowding, for before segregation was well established in hospitals, upwards of 60 o/o of the patients in one or two places showed streptococcus haemolyticus in their throats.

The accompanying charts, constructed from the weekly reports of pneumonia and measles from Base Hospitals, (which have been published in the *Journal of the A. M. A.*) serve to give some idea of the incidence and distribution of pneumonias in a few selected camps, and cantonments.

The warning which this epidemic in the United States gives to us in the A. E. F. is obvious. Evidently men who harbor streptococcus haemolyticus are constantly reaching France, and though it is to be hoped that no such epidemic will occur here as affected the recruits, still it is probable that individuals suffering from certain types of disease will be found highly susceptible to infection. It is expected that the troops that arrive here are largely immune to measles, and that therefore little trouble should be experienced from this disease.

The influenza, however, is already being complicated by pneumonia, and it is very possible that streptococcus haemolyticus will play an important part as a complicating factor in this disease.

The gassed cases, too, are highly susceptible to secondary infection of the respiratory tract and utmost care should be taken to protect them from cases suffering from streptococcus infection, or, if possible, from carriers.

## Figures and Tables

**Figure f1:**
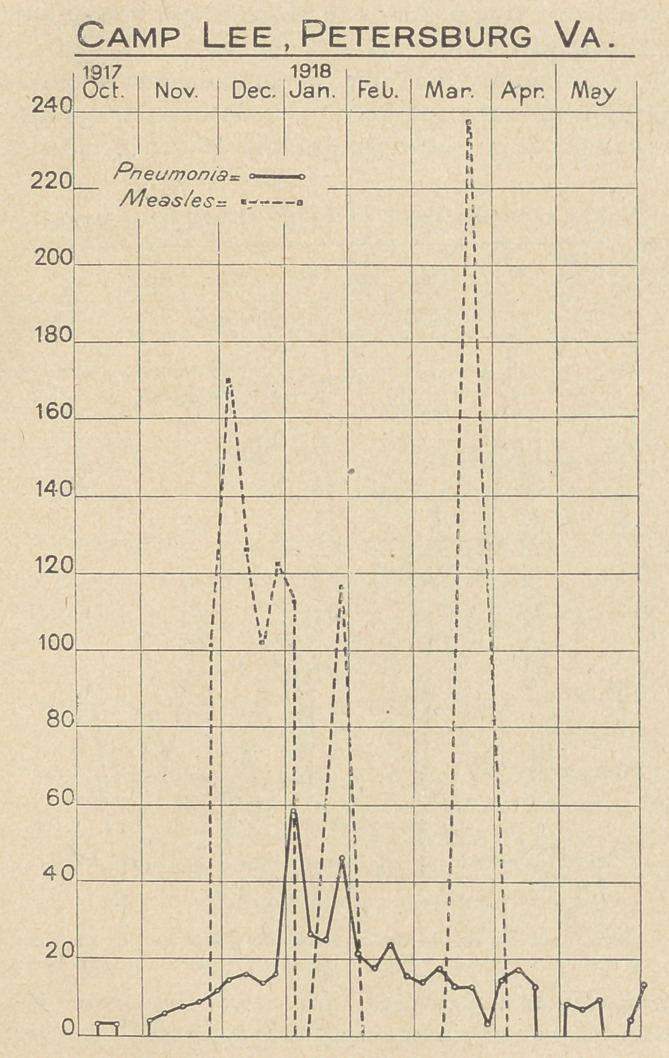


**Figure f2:**
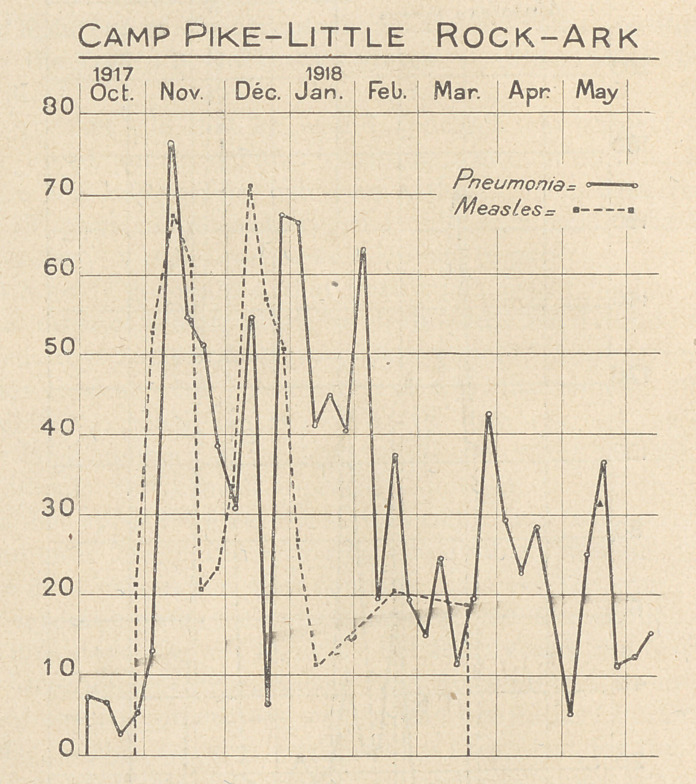


**Figure f3:**
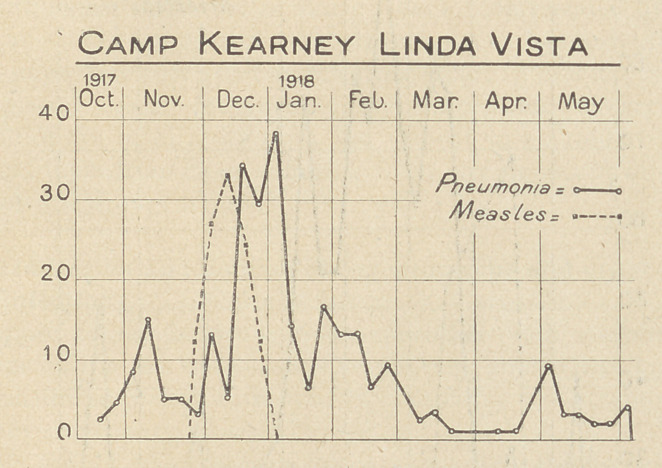


**Figure f4:**